# Genetic polymorphisms associated with treatment failure and mortality in pediatric *Pneumocystosis*

**DOI:** 10.1038/s41598-018-38052-x

**Published:** 2019-02-04

**Authors:** Yogita Singh, Bijay Ranjan Mirdha, Randeep Guleria, Sushil K. Kabra, Anant Mohan, Rama Chaudhry, Lalit Kumar, Sada Nand Dwivedi, Sanjay K. Agarwal

**Affiliations:** 1 All India Institute of Medical Sciences, Department of Microbiology, New Delhi, 110029 India; 2 All India Institute of Medical Sciences, Department of Pulmonary Medicine and Sleep Disorders, New Delhi, 110029 India; 3 All India Institute of Medical Sciences, Department of Pediatrics, New Delhi, 110029 India; 4 All India Institute of Medical Sciences, Department of Medical Oncology, New Delhi, 110029 India; 5 All India Institute of Medical Sciences, Department of Biostatistics, New Delhi, 110029 India; 6 All India Institute of Medical Sciences, Department of Nephrology, New Delhi, 110029 India

## Abstract

Data on the genetic diversity of *Pneumocystis jirovecii* causing *Pneumocystis* pneumonia (PCP) among children are still limited, and there are no available data from the Indian subcontinent, particularly associations between genotypes and clinical characteristics. A total of 37 children (62 days-12 years [median 5.5 years]) were included in this study. Pneumocystis was diagnosed by microscopy using Grocott-Gomori methenamine silver stain in 12 cases and by nested PCR using *mtLSUrRNA* in 25 cases. Genotyping was performed using three different genes, mitochondrial large subunit ribosomal RNA (*mtLSUrRNA*), dihydropteroate synthase (*DHPS*) and dihydrofolate reductase (*DHFR*). *mtLSUrRNA* genotype 3 and novel mutations at the gene target *DHFR* (401 T > C) and *DHPS* 96/98 were frequently observed and clinically associated with severe PCP and treatment failure. Phylogenetic analyses revealed 13 unique sequence types (STs). Two STs (i) *3-DHFR 401 T* > *C-DHPS 96/98* – PJ1 and (ii) *3-DHFR 401 T* > *C-DHPS 96*- PJ3 were significantly associated with treatment failure and high mortality among PCP-positive patients. In conclusion, the present study strongly suggests the emergence of virulent *P*. *jirovecii* strains or genetic polymorphisms, leading to treatment failure and high mortality. Our study is the first of its kind from the Indian subcontinent and has highlighted the genetic diversity of *Pneumocystis jirovecii* among children and their clinical outcomes. These findings emphasize the need to focus more on genotypes to better understand the epidemiology of *Pneumocystis* pneumonia.

## Introduction

*Pneumocystis jirovecii* (*P*. *jirovecii*), an opportunistic pathogen, causes life-threatening *Pneumocystis* pneumonia (PCP), mostly among immunocompromised individuals. *Pneumocystis* pneumonia, earlier termed interstitial plasma cell pneumonia, established itself as a clinical entity shortly after World War II when it was diagnosed as one of the causes of debilitating pneumonia in severely malnourished and premature infants^1,2^. In developing countries, *Pneumocystis* pneumonia is one of the important causes of respiratory morbidity, leading to significant mortality among children with AIDS and accounting for 10–40% or higher of deaths^3–5^. Molecular characterization of *P*. *jirovecii* is still in an evolving mode worldwide and has become a topic of immense research, although some studies from different parts of the globe have shown to some extent an association between genetic diversity, clinical characteristics and disease outcome, particularly in children^6,7^.

Among the various molecular techniques used to date for *Pneumocystis jirovecii*, multilocus sequence typing (MLST) has often been considered the gold standard to study its population structure^8–11^. Various studies conducted in this regard have used both coding and noncoding regions of *P*. *jirovecii* as the gene targets to delineate genetic polymorphism(s) within the organism and its clinical associations^9,11–13^. Earlier studies on genetic characterization have shown that *P*. *jirovecii* is heterogeneous in distribution, i.e., genetically different strains of this organism are circulating worldwide, and these strains have associations with pathogenicity and other diverse clinical manifestations^9,14–18^. Studies have also shown that specific dihydropteroate synthase (*DHPS*) gene mutations or genotypes of *P*. *jirovecii* were associated with decreased efficacy of sulfa treatment and disease severity^19^.

In the present study, sequence typing of *P*. *jirovecii* was conducted using *P*. *jirovecii*-specific loci, such as mitochondrial large subunit ribosomal RNA (*mtLSUrRNA*), dihydropteroate synthase (*DHPS*) and dihydrofolate reductase (*DHFR*) genes, to unfold genetic diversity or polymorphism(s) in clinical samples obtained from pediatric patients from the Indian subcontinent. MLST schemes using these gene targets were selected with a rationale that in our tertiary care hospital, we could observe severe pneumocystosis and high mortality in pediatric patients despite administration of the full course of anti-*pneumocystis* treatment. Hence, it was planned to extrapolate gene polymorphisms or genetic variations that are circulating among our pediatric population and have any bearing with the clinical presentations as well as in the disease outcome. The present study is the first of its kind from India because data on genetic diversity and their clinical association among pediatric populations are not available until now.

## Results

### Demographic and clinical characteristics of pediatric patients

A total of thirty-seven (37/190; 19.4%) samples were positive for *P*. *jirovecii* by nested PCR assay targeting the *mtLSUrRNA* gene (only 12 samples were positive by microscopic examination, 11 samples from bronchoalveolar lavage fluid (BALF) and 1 sample from Sputum). These 37 pediatric patients (age ranging from 0.17 years to 12 years) included five HIV-infected children, ten patients with different malignant disorders, six patients with autoimmune disorders and immune deficiencies and sixteen patients from another group. These latter 16 patients had different kind of underlying diseases (had symptoms of pneumonia) and were not among the study population that had a diagnosis of HIV infection, malignancy, autoimmune disorders or immune deficiencies. These 16 patients were categorized as “others” (details of each case shown in Tables 1 and 2). Samples from twenty-five patients (25/37;68%) were microscopy-negative; however, these patients were considered true-PCP cases because each of them had clinical features highly suggestive of *Pneumocystis* pneumonia at the time of hospital admission. Upon further analyses, it was observed that 27 patients had intensive care unit (ICU) admissions and 25 (92.5%) of them required mechanical ventilation. Twenty-seven patients (27/37; 73%) had mild hypoxemia with partial pressure of oxygen (PaO_2_) less than 95%, and 10 patients had severe hypoxemia with PaO_2_ less than 60%. Twelve out of 37 PCP patients (32%) had coinfections with bacterial and viral pathogens, as shown in Tables 3 and 4. After confirmed laboratory diagnosis of PCP, all positive pediatric patients were given a combination of anti-*pneumocystis* treatment (TMP-SMX), except in one patient, where TMP-SMX was given initially, but later, the treatment was switched to intravenous clindamycin due to deranged liver function. Thirty-one patients (84%) had less than four weeks (<4 weeks) of hospital stay, whereas 6 patients (16.2%) had more than four weeks (>4 weeks) of hospital stay. Despite anti-*Pneumocystis* treatment, 17 patients (45.9%) had fatal outcomes due to hypoxemic pneumonia, which included 12 microscopy-positive samples and five of the 25 testing positives by nPCR assay only. Among these 17 patients with negative outcomes, nine patients had coinfections with bacterial or viral pathogens (*P*-value = 0.069). All survival cases were followed-up for 4 weeks after discharge to confirm their progress of recovery.

**Table 1:** *Pneumocystis jirovecii* Genotypes and Sequence Types.

patients ID	Age (year)^~^	Underlying Conditions^$$^	ICU admission	Mechanical ventilation	Hypoxia″	coinfections^##^	Response*	GMS^**^	*mtLSUrRNA*	*DHFR* ^*#*^	*DHPS 96* ^*$*^	*DHPS 98* ^*@*^	*Sequence Types*
1	0.17	Persistent pneumonia	YES	YES	SEVERE	PA	E	N	3	M	M	M	PJ1
2	0.17	LRTI	YES	YES	SEVERE	NO	S	N	2	M	M	M	PJ2
3	0.17	ARDS	YES	YES	SEVERE	KP	E	N	3	M	M	WT	PJ3
4	0.25	PTB	YES	YES	SEVERE	PTB	E	Y	3	M	M	M	PJ1
5	0.25	Pneumonia	YES	YES	SEVERE	NO	S	N	3	WT	M	M	PJ4
6	0.33	Pneumonia	YES	YES	SEVERE	NO	E	Y	3	M	M	M	PJ1
7	0.58	Pneumonia	YES	YES	SEVERE	NO	E	Y	2	M	M	M	PJ2
8	0.92	Pneumonia	YES	NO	SEVERE	NO	E	Y	1	WT	M	WT	PJ5
9	1	AML	NO	YES	MILD	NO	S	N	1	M	M	M	PJ6
10	2	ARDS	YES	NO	SEVERE	NO	E	Y	2	M	WT	M	PJ7
11	4	ALL	YES	YES	SEVERE	NO	S	N	2	M	M	WT	PJ8
12	4	ALL	NO	NO	MILD	NO	S	N	1	M	M	M	PJ6
13	4	PTB	YES	YES	SEVERE	PTB	E	Y	3	M	M	M	PJ1
14	5	HIV/Persistent pneumonia	NO	NO	MILD	PTB	S	N	1	WT	WT	WT	PJ9
15	5	Pneumonia/PTB	YES	YES	SEVERE	PTB/CMV	E	N	3	M	M	WT	PJ3
16	5	AML	YES	YES	SEVERE	NO	E	Y	3	M	M	M	PJ1
17	5	HIV/PTB	YES	YES	SEVERE	PTB	E	Y	2	WT	WT	WT	PJ10
18	6	HIV/PTB	YES	YES	SEVERE	PTB	E	Y	3	M	M	WT	PJ3
19	7	CVID	NO	NO	MILD	NO	S	N	3	WT	M	M	PJ4
20	7	ALL	YES	YES	SEVERE	NO	E	Y	1	M	M	WT	PJ11
21	8	ALL	NO	NO	MILD	NO	S	N	3	WT	M	WT	PJ12
22	9	LRTI	YES	YES	SEVERE	NO	E	Y	3	M	M	WT	PJ3
23	10	ALL	YES	NO	MILD	NO	S	N	1	M	M	M	PJ6
24	11	HIV	NO	NO	MILD	NO	S	N	2	WT	WT	WT	PJ10
25	11	LRTI	YES	YES	SEVERE	NO	E	Y	2	M	M	M	PJ2
26	12	HIV/PTB	NO	NO	MILD	PTB	S	N	1	WT	WT	WT	PJ9
27	12	ALL	NO	NO	MILD	NO	S	N	2	WT	WT	WT	PJ10
28	12	ALL	NO	NO	MILD	NO	S	N	2	WT	WT	WT	PJ10
29	12	Pneumonia	YES	YES	MILD	NO	S	N	3	M	WT	WT	PJ13
30	12	Pneumonia	YES	YES	MILD	NO	S	N	1	WT	WT	WT	PJ9

^~^Age: in years (days and months were also converted into year).

*Response: disease outcome; E: expired; S: survival; ^*#*^*DHFR* M: mutation at 401 position (T to C); ^$^*DHPS 96 *M: mutation at codon 96 (288; G to A); ^@^*DHPS* 98 M: mutation at codon 98 (294; G to C); WT: wild type; ″Hypoxia Severe PaO_2_ < 60%; Mild PaO_2_ < 95%; PTB: pulmonary Tuberculosis; ARDS: Acute respiratory distress syndrome; LRTI: Lower respiratory tract infections; CVID: common variable immunodeficiency; ^$$^Underlying conditions: conditions with patients presented or had it at the time of admission;

**GMS: microscopic examination by Grocott-Gomori methenamine silver staining method; Y: positive; N: negative; ^##^Coinfections (along with PCP): PA: *Pseudomonas aeruginosa;* KP: *Klebsiella*. *pneumoniae*; PTB: pulmonary tuberculosis caused by *M*. *tuberculosis;* CMV: *Cytomegalovirus* pneumonia; ALL: Acute lymphoblastic leukemia; AML: Acute myeloid leukemia.

**Table 2:** Brief details of patients positive for PCP by *mtLSUrRNA* PCR assay and negative by *DHFR*, *DHPS* PCR assay and microscopic examination.

S.No.	Age (yr)/sex**	Underlying conditions*	Respiratory sample	Fever	Cough	Dyspnea	CXR/CT-scan^$^	Coinfections	Response^@^
1	0.08/M	Pneumonia	BALF	Yes	Dry	Yes	B/l infiltrates/GGO	*M*. *tuberculosis*	S
2	1.5/M	Malnutrition/Pneumonia	Sputum	Yes	Dry	Yes	B/l infiltrates/GGO	No	S
3	2/M	Cystic fibrosis/pneumonia	BALF	No	Dry	Yes	B/l infiltrates/GGO	*Ps*. *aeruginosa*	E
4	4.5/M	Interstitial lung disease	Sputum	No	Expectorated	Yes	B/l infiltrates/NA	No	S
5	5/M	Sarcoidosis	BALF	Yes	No cough	No	B/l hilar lymphadenopathy/NA	No	S
6	5.5/M	Pneumonia	BALF	Yes	Dry	Yes	B/L infiltrates/NA	No	S
7	7.42/M	Lymphoproliferative syndrome with severe pneumonia	BALF	Yes	Dry	Yes	B/L infiltrates/GGO	*M*. *tuberculosis*	E

**Age given in years; M: Male; F: Female.

*Underlying Conditions: Presenting conditions.

^$^CXR: chest X-ray; CT-Scan: computed tomography; B/L infiltrates: bilateral perihilar infiltrates; GGO: ground glass opacities; NA: not available.

^@^E: expired; S: survival.

**Table 3:** Mutations and their clinical significance.

Clinical variables		DHFR genotypes			DHPS genotype			DHPS genotype		
		WT	401	*p*-value	WT	Val96Ile*	*p-*value	WT	Glu98Gln*	*p*-value
HIV-seropositive (n = 5)		4	1	**0**.**047**	4	1	**0**.**019**	5	0	**0**.**045**
Malignant disorder (n = 9)^~^		3	6	>0.99	2	7	0.681	5	4	>0.99
Autoimmune disorder and immune-deficiencies (n = 5)^~^		2	3	>0.99	1	4	>0.99	3	2	>0.99
Other group^$^ (n = 11)		2	9	0.140	2	9	0.419	3	8	0.057
ICU (n = 21)		5	17	**0**.**02**^**#**^	4	17	0.08	10	11	0.4
Severe hypoxia (Pa0_2_ < 60 mmHg) (n = 17)		4	14	(0.06)	2	15	**0**.**01**^**#**^	7	10	0.15
Mechanical ventilation (n = 19)		4	16	**0**.**01**^**#**^	3	16	**0**.**04**^**#**^	9	10	0.46
Coinfections (n = 9)		3	6	0.109	3	6	0.109	6	3	0.440
Response	Non-Survivors (n = 15/30)	2	13	**0**.**021**^**#**^	2	13	0.06	7	8	0.71
	Survivors (n = 15)	9	6		7	8		9	6	

^#^*p*-value: significant at value < 0.05; WT: wild type; Val96Ile* and Glu98Gln*: DHPS mutation at codon 96 and 98. ^$^Other group: patients presented with lower respiratory tract infections; persistent pneumonia; acute respiratory distress syndrome; pulmonary tuberculosis; asthma (on Steroids); interstitial lung disease (ILD). Coinfections: *M*. *tuberculosis*, *Klebsiella pneumoniae*, *Pseudomonas aeruginosa*, *Cytomegalovirus* pneumonia; ^~^malignant disorders included acute lymphoblastic leukemia (ALL), acute myeloid leukemia (AML), lymphoproliferative disorder; ^~^autoimmune disorders and immune deficiencies include sarcoidosis, steroid-resistant nephrotic syndrome (SRNS), common variable immune deficiency (CVID), chronic granulomatous disease (CGD).

**Table 4:** Clinical and genotypic associations.

Clinical and genotypic variables	Survivors	Nonsurvivors	Frequency/*P*-value
HIV-seropositive (n = 5)	3	2	1
Malignant disorders (n = 9)^~^	7	2	0.109
Autoimmune disorders and immune-deficiencies (n = 5)^~^	2	3	1
Other group^$^ (n = 11)	3	8	0.128
ICU (n = 21)	6	15	0.001
Severe hypoxia (Pa0_2_ < 60 mmHg) (n = 17)	3	14	<0.0001
Mechanical ventilation (n = 19)	6	13	0.021
Coinfections (n = 9)	2	7	0.109
**DHFR:**			
WT	9	2	0.021
401 T > C	6	13	
**DHPS (Val96Ile)**			
WT	7	2	0.06
Val96Ile*	8	13	
**DHPS (Glu98Gln)**			
WT	9	7	0.715
Glu98Gln*	6	8	

^~^malignant disorders included acute lymphoblastic leukemia (ALL), acute myeloid leukemia (AML), lymphoproliferative disorder; ^~^autoimmune disorders and immune deficiencies included sarcoidosis, steroid-resistant nephrotic syndrome (SRNS), common variable immune deficiency (CVID), chronic granulomatous disease (CGD). ^$^Other group: patients presented with lower respiratory tract infections; persistent pneumonia; acute respiratory distress syndrome; pulmonary tuberculosis; asthma (on Steroids); interstitial lung disease (ILD). Val96Ile* and Glu98Gln*: DHPS mutation at codons 96 and 98.

### *Pneumocystis* genotypes and its clinical association

#### mtLSUrRNA genotyping

Among 37 pediatric patients, genotype 1 was observed in 13 patients (mt85C; 35%), followed by genotype 3 in 13 patients (mt85T; 35%) and genotype 2 in 11 patients (mt85A; 30%). Furthermore, genotype 3 was frequently associated with patients who had severe episodes of *Pneumocystis* pneumonia and fatal outcomes (9/17; *P-*value = 0.04). Considering the underlying conditions shown in Table 1, genotype 3 was the most virulent genotype and was most frequent in patients who presented with severe pneumonia in comparison to other underlying conditions (HIV-seropositive, malignant disorders, autoimmune disorders and immunodeficiencies; *P*-value = 0.641).

#### *DHFR* genotyping

A total of 30 samples from patients (30/37; 81%) were amplified using this locus by a nested PCR assay. Of these 30 samples, 19 samples had nonsynonymous nucleotide substitutions at position 401 (T to C), and the remaining 11 samples were infected with the wild-type strain.

This mutation led to an amino acid change from valine to alanine. Clinically, this nucleotide polymorphism was frequently observed in patients who had a severe form of the disease (Table 3), undergone ICU admissions (17/30; *P*-value = 0.02), had PaO_2_ less than 60 mmHg (14/30; *P*-value = 0.06)] and required mechanical ventilation (16/30; *P*-value = 0.01). This mutant strain (401 T > C) was responsible for the rapid deterioration of clinical disease, and most of these infected patients (10/17; 56%) died within four weeks of PCP infection. Regarding the treatment aspects, we observed that this nonsynonymous mutation led to treatment failure and death in 13 (68%; *P*-value = 0.021) of the infected children. It was also observed that the *DHFR* mutant strain (401 T > C) was frequently associated with patients presenting with pneumonia (other group; 9/11, 81%), followed by 66% in patients with malignant disorders, 60% in patients with autoimmune disorders or immune deficiencies and 20% in patients with HIV seropositivity (Table 3). Nine patients (9/30; 30%) had coinfections (Tables 1 and 4), of which, six patients had both coinfections and infection with a mutant strain of *P*. *jirovecii* (*P-*value = 0.109). These combinations might be responsible for rapid deterioration with a fatal outcome.

#### *DHPS* genotyping

The *DHPS* gene could also be amplified in only 30 PCP-positive samples using a nested PCR assay. At this locus, novel mutations at nucleotide positions 288 and 294 were observed, which were different from all the mutations reported in the literature thus far. The most common mutations reported worldwide are at codons 55 (nucleotide position 165) and 57 (nucleotide position 171). Mutations that were observed in the present study at nucleotide positions 288 (G to A) and 294 (G to C) were nonsynonymous, resulting in an amino acid change from valine to isoleucine at codon 96 (Val96Ile) and glutamic acid to glutamine at codon 98 (Glu98Gln), respectively. Overall, in the present study, we observed three novel DHPS mutations or genetic variations: mutant 96 (21/30; 70%); mutant 98 (14/30; 47%) and double mutant 96/98 (13/30; 43%).

Mutant 96 (genotype 288; G to A) was associated with severe PCP most frequently in patients from the “other group” (presented with severe pneumonia; 9/11, *P*-value = 0.057) and in patients with autoimmune disorders and immune deficiencies (4/5; Table 3). Seventeen patients with this mutant strain had ICU admissions (*P*-value = 0.08), 16 patients required support for mechanical ventilation (*P*-value = 0.04), 15 patients had severe hypoxia, i.e., PaO_2_ less than 60 mmHg (*P*-value < 0.0001), and 13 patients had fatal outcomes (*P-*value = 0.06). As observed with the *DHFR* mutant strain, the same number of patients (9/30) infected with the *DHPS* mutant strain also had coinfections (*P-*value = 0.109; Tables 1, 3 and 4).

Mutant 98 (genotype 294; G to C) was also associated with severe PCP, most frequently in patients from the “other group” (presented with severe pneumonia; 8/11, *P*-value = 0.057) (Table 3). Eleven out of 30 patients had ICU admissions (*P*-value = 0.4), of which 10 patients required mechanical ventilation (*P-*value = 0.4) and 8 out of them had fatal outcomes (*P*-value = 0.7). Double mutant Val96Ile/Glu98Gln was present in 13 patients, and 7 of them had anti-*pneumocystis* treatment failure and had fatal outcomes in comparison to only one death due to the wild-type DHPS strain (*P*-value = 0.009).

### Multilocus sequence typing, phylogenetic analyses and its clinical association

**Figure 1:**
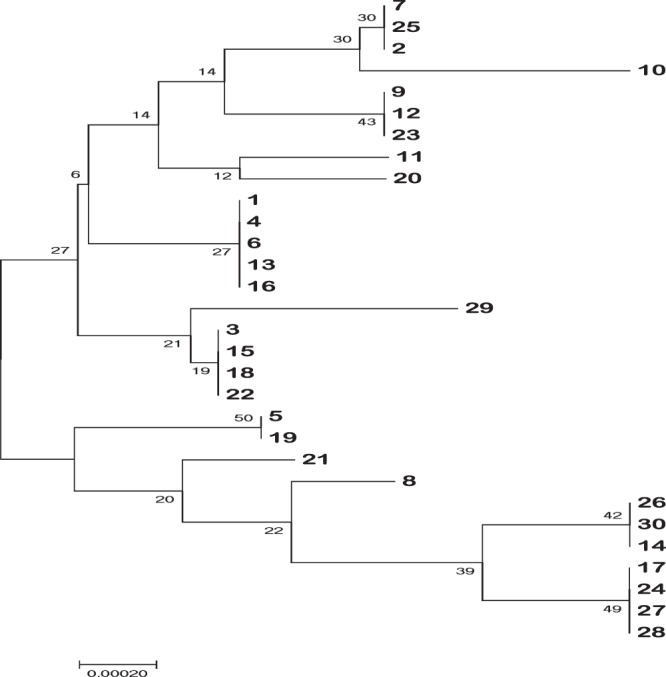
Phylogenetic analyses of *P*.*jirovecii*: Phylogenetic tree was inferred using Neighbor-Joining method. The evolutionary distances were computed using the Kimura 2-parameter method and involved 30 concatenated nucleotide sequences showing 13 unique sequence types from PJ1 to PJ13 (shown as 1 to 13 respectively).

Multilocus sequence typing could be performed in 81% of samples (30/37) using all three gene targets. Upon phylogenetic analyses, thirteen unique heterogeneous sequence types (STs) were obtained in the study cohort and were subsequently named PJ1 to PJ13 (Fig. 1; Table 1). Considering *mtLSUrRNA*, *DHFR*, and *DHPS* typing, two STs, (i) *3-DHFR 401 T* > *C-DHPS 96/98* **(PJ1)** (5/30;16.7%) and (ii) *3-DHFR 401 T* > *C-DHPS 96* **(PJ3)** (4/30; 13.3%), were more frequent in comparison to other STs in the present study. Clinically, these two STs were associated with PCP patients who had ICU admission, had PaO_2_ less than 60 mmHg, required mechanical ventilation and had fatal outcomes (*P-*value = 0.009 for PJ1; *P*-value = 0.02 for PJ3) (Table 1).

## Discussion

PCP in the pediatric group is commonly seen in infants that are premature and malnourished, children on immunosuppressive therapy that have undergone transplantation, children with primary immunodeficiency disorders and children infected with HIV^20^. Recently, *P*. *jirovecii* infection has also been considered a risk factor for high mortality among children^5^. A study performed on Mozambican children showed that PCP seemed to be associated with worsening disease in comparison to non-PCP children^21^. Molecular characterization of *P*. *jirovecii* and its associated genotypes with clinical characteristics has been a matter of intense research to strategize an effective method to control the disease.

In the present study, we performed multilocus sequence typing targeting unique combinations of genes, including a gene involved in metabolic functions (*mtLSUrRNA* gene) and two enzymatic targets (genes) of therapeutic agent trimethoprim/sulfamethoxazole (*DHFR* and *DHPS*). Only 12 patients were positive by microscopic examination (GMS) out of 37 patients who were positive by *mt LSU rRNA* nested PCR assay. All microscopy-positive PCP cases had fatal outcomes. The remaining 25 cases were clinically documented cases of PCP, as all these cases had a high index of clinical suspicion of *Pneumocystis* pneumonia at the time of presentation and further supported by a positive PCR assay. In this context, studies have previously reported that microscopy-negative PCP cases may also be addressed as possible or probable cases under the following conditions: (i) if clinical findings are consistent with PCP, (ii) if they are positive by PCR (*mtLSUrRNA*) assay, and (iii) if resolution of symptoms with empiric anti-*Pneumocystis* therapy observed^22–24^. Furthermore, of these 25 clinically documented cases of PCP, 54% (20/37) patients responded to anti-*Pneumocystis* treatment, and 13.5% (5/37) succumbed to death because of severe pneumonia resulting in respiratory failure. Moreover, nested PCR assays targeting *mtLSUrRNA* for PCP diagnosis have been considered more sensitive than microscopic examinations performed by GMS staining or single-step PCR assays if the patients were clinically symptomatic, i.e., clinically documented PCP cases^25–29^. In the context of colonization, a recently published study from Poland, performed on *Pneumocystis* colonized cases, stated that *P*. *jirovecii-*colonized patients were defined as individuals who did not have clinical symptoms or radiological signs of PCP^30^.

The overall prevalence of PCP among children was 19.4%, with 45.9% mortality, which is relatively greater than that reported in our earlier study of 15% prevalence, with 21.4% mortality^31^. By stratifying the underlying disease category included in the present study, it was observed that the prevalence of PCP was 40% in patients with a diagnosis of autoimmune disorders and with immune deficiencies followed by 25% in patients with malignant disorders, 23.8% in HIV-seropositive patients, and 16% in patients with severe pneumonia (Table 4). Considering underlying disease severity and PCP as one of the important antecedent causes of death, the highest mortality was observed among patients who presented with severe pneumonia (9/16; 56.25%), followed by 50% mortality among patients with autoimmune disorders and with immune deficiencies (3/6), 40% in patients with HIV (2/5), and 30% among patients with malignant disorders (3/10).

Worldwide, *mtLSUrRNA* genotypes 2 and 3 have been associated with severe *Pneumocystis* pneumonia and have fatal outcomes^32,33^. Genotype 3 was the most frequent genotype among all enrolled patients. We observed a significant association between genotype 3 and PCP patients with fatal outcomes in the study population (9/17; *P-*value = 0.04).

*DHFR* genotyping revealed a previously unreported mutation at single nucleotide position 401 (T > C) that resulted in the substitution of an amino acid (V134A). This mutation was significantly associated with severe episodes of *Pneumocystis* pneumonia, and 13 out of 19 pediatric patients infected with this mutant strain had treatment failure and succumbed to PCP (*P-*value = 0.021). The *DHFR* gene is the therapeutic target of trimethoprim (TMP), a part of combinations of trimethoprim/sulfamethoxazole (TMP-SMX) used for the treatment of PCP. A point mutation in the coding region of this gene can lead to alteration in the amino acid, resulting in drug resistance in *P*. *jirovecii*^34,35^ as well as in other microorganisms^36–39^. This nonsynonymous point mutation can occur because of selective pressure of the drugs at this nucleotide position, or it might be a genetically different (novel genetic variation) strain circulating among our population exerting more a virulent effect on infected patients. However, the effect of this single nucleotide polymorphism at the protein level could not be delineated.

*DHPS* genotyping showed a novel point mutation (nonsynonymous) at two amino acid codons (Val96Ile/Glu98Gln). The most common point mutations responsible for drug resistance to sulfa drug (anti-*pneumocystis* drug; SMX) reported worldwide, i.e., Thr55Ala and Pro57Ser and another nonsynonymous mutation, Asp90Asn and Glu98Lys^40–42^, in the *P*. *jirovecii* strain were not detected in our study group. In our study, patients with DHPS mutations 96 (Val > Ile) were associated with severe hypoxia (*p-*value = 0.01) during PCP episodes and required mechanical ventilation (*p-*value = 0.04). Thirteen out of 21 patients harboring this mutation (Val96Ile) also had fatal outcomes. Patients infected with both DHPS mutations together (double mutant Val96Ile/Glu98Gln) were frequently associated with no response to anti-*pneumocystis* treatment (TMP-SMX). The possible reason behind the occurrence of these point mutations could be attributed as the same as mentioned for *DHFR* gene mutations observed in the present study. In addition to these findings, we observed that mutations or genetic variations in our study (Val96Ile and Glu98Gln) were in the same conserved region (in the same coding regions) as those mutations that have been described previously for drug resistance (Thr55Ala and Pro57Ser) in *Pneumocystis jirovecii*^40^. Hence, it is strongly suggested that these novel mutations may also exert similar drug resistance effects. Similar findings had been reported by a study from Santiago, Chile, where authors suggested that patients infected with *DHPS* mutants required a twice-longer duration of mechanical ventilation and showed decreased efficacy of TMP-SMX. However, they did not find any significant associations between specific *DHPS* genotypes/mutations and mortality^19^.

*DHFR* and *DHPS* genes are single-copy genes and could not be amplified in 7 out of 37 samples, which was one of the limitations of our study. This result could be because of the low load of *P*. *jirovecii* in these clinical samples. Amplification failure at a single copy gene in comparison to a multi copy-gene, such as *mtLSUrRNA*, due to low fungal burden, particularly in HIV-negative immune-compromised patients, has been reported by several authors^34,43,44^.

Multilocus sequence typing showed 13 unique STs (PJ1 to PJ13). The most frequent STs were *3-DHFR 401 T* > *C-DHPS 96/98* and *3-DHFR 401 T* > *C-DHPS 96*. These two STs were considered more virulent or pathogenic, as these were associated with high mortality among PCP-proven cases (PJ1, *P* = 0.009; PJ3, *P* = 0.02), especially among patients presented with severe pneumonia in comparison to other underlying causes (HIV-seropositive, malignant disorders, autoimmune disorders, immune-deficiencies). In further analyses, we observed that wild type genotypes (*DHFR*, *DHPS*) were associated with HIV-seropositive patients (*P*-value < 0.05), indicating concomitant severity of underlying conditions and coinfections in these patients (4 out of 5 PCP patients with HIV-seropositivity had coinfections with *M*. *tuberculosis* and 2 of them died).

Overall, infection with the *Pneumocystis jirovecii* mutant strain was an important antecedent cause of disease severity and death; however, there were a total of 9 non-surviving (9/37; 24%) patients with coinfecting pathogens (Tables 1, 2 and 4), which were simultaneously responsible for disease severity and rapid deteriorations among these fatal cases (*P*-value = 0.109). PCP with coinfections has been reported earlier with a significant increase in mortality rate, especially among children less than 5 years old and HIV-seropositive patients with CD4+ T-cell counts less than 200 cells/µl. Pulmonary coinfections, including *Pneumocystis jirovecii* and *M*. *tuberculosis*, have been reported earlier and are known to facilitate disease severity and poor outcome. Similarly, combinations of *Pneumocystis jirovecii* and CMV in immunocompromised patients have been reported to be life-threatening^21,45^.

In conclusion, our observations suggest that the occurrence of these mutations or genetic variations in *P*. *jirovecii* together can be considered virulent or more pathogenic strains. Infection by these mutant strains can lead to severe PCP, drug resistance and/or treatment failure. Coinfecting pathogens should always be monitored simultaneously with *P*. *jirovecii*-infected in children, as coinfections mostly results in poor outcomes. An effective alternate anti-*pneumocystis* treatment should always be considered well in time in the case of nonresolution of symptoms due to *Pneumocystis* pneumonia. In this context, it is proposed that research from different parts of the world, including a large number of patients, may reveal more of these mutations and sequence types to reinforce the genotype association with clinical variability and disease outcome.

## Materials and Methods

### Subjects

One hundred ninety (n = 190) children (0.08 years to 12 years of age) who attended to both out-patient and in-patient departments of our tertiary care hospital were included prospectively during the study period of three years from February 2014 to March 2017. BALF (n = 140) and sputum (n = 50) were collected before the start of the anti-*Pneumocystis* treatment. These children comprised both human immune-deficiency virus (HIV)-infected children (n = 21) and children who had infections other than HIV (n = 169) (details of non-HIV patients are given in Table 5). These patients were clinically suspected cases of *Pneumocystis* pneumonia (PCP) and had at least had two of the typical features suggestive of PCP, such as fever, unproductive cough, and dyspnea, at the time of enrollment with suggestive radiological findings, such as chest radiographs showing diffuse bilateral peri-hilar infiltrates or CT scan showing ground-glass opacities. Various relevant clinical details were recorded (Table 5). A follow-up study was conducted in all patients. “Positive follow-up” was considered when the PCP-positive patients responded to the anti*-pneumocystis* treatment (trimethoprim/sulfamethoxazole; TMP-SMX) and survived for at least four weeks after recovery from the disease. However, “negative follow-up” was considered when the PCP-positive patient failed to respond to anti-*pneumocystis* treatment and had a fatal outcome. None of the PCP-positive patients had prior exposure to sulfamethoxazole-trimethoprim or a past history of PCP.

**Table 5:** Clinical characterization of pediatric patients enrolled in the study.

Patients details	Total number of patients enrolled (n = 190)	Total number of PCP negative cases (frequency, %)	Total number of PCP positive cases (frequency, %)
**Sex**			
Male	118	94 (79.7)	24 (20.3)
Female	72	59 (82)	13 (18)
**Clinical samples**			
BALF^#^ (%) (n = ^$^)	140	111 (79)	29 (21) (n = 11)
Sputum (%) (n = ^$^)	50	42 (84)	8 (16) (n = 1)
**Underlying conditions**			
HIV-infected	21	16 (76.2)	5 (23.8)
Malignant disorders	40	30 (75)	10 (25)
Autoimmune disorders and immune-deficiencies	15	9 (60)	6 (40)
Others*	114	98 (86)	16 (14)
**Clinical features present at the time of enrollment**			
Fever	170	134 (79)	36 (21)
Dyspnea	140	107 (76.4)	33 (23.6)
Cough	112	55 (69.6)	24 (30.3)
Nonproductive	79	24 (72.8)	9 (27.2)
Expectoration	33		
**Supporting Radiological findings** ^**^			
Chest X-ray	50	19 (38)	31 (62)
CT Scan	40	24 (60)	16 (40)
**CD4+ count (only for HIV positives cases):**			
<350 cells/ul	21	16 (76.2)	5 (23.8)
>350 cells/ul	19 2	14 (73.7) 0	5 (26.3) 0
**Response**			
Survival	164	144	20 (54)
Death	26	9^@^	17 (46)

*Others: patients presented with lower respiratory tract infections; persistent pneumonia; acute respiratory distress syndrome; pulmonary tuberculosis; asthma (on Steroids); interstitial lung disease (ILD).

**Chest X-ray: B/L diffuse infiltrates; CT-Scan: Ground Glass opacities (GGO).

^#^BALF: Bronchoalveolar lavage fluid; %: frequency

^$^n = number of samples positive by microscopic examination (GMS).

^@^death due to underlying disease severity or pneumonia other than PCP.

This study was ethically approved by the All India Institute of Medical Sciences, New Delhi Ethics Committee **(Institutional Ethics Committee-IESC/T-77)**. All enrolled patients were given a detailed appraisal about the objective of the study.

### Informed consent was obtained from a parent and/or legal guardian for study participation

All the methods used in the present study were carried out in accordance with the relevant guidelines and regulations.

Respiratory specimens, including bronchoalveolar lavage fluid (BALF; n = 140) and sputa (n = 50) (where BAL was not possible), were collected from enrolled patients. Laboratory investigations for detecting *P*. *jirovecii* were performed using both direct microscopy (Grocott-Gomori methenamine silver staining) and nested polymerase chain reaction (nPCR) assays targeting the mitochondrial large subunit ribosomal RNA (*mtLSUrRNA*) gene^46,47^. The presence of any other concurrent infections (bacterial, viral and fungal) was studied using recommended microbiological cultures and/or PCR assays. For bacterial speciation, MALDI-TOF (matrix-assisted laser desorption ionization-time of flight) was used.

### Genotyping

Genotyping of *P*. *jirovecii* was carried out using three loci, *mtLSUrRNA*, dihydrofolate reductase (*DHFR)* and dihydropteroate synthase *(DHPS)* genes.

A nested PCR assay was performed for all three gene loci. The PCR assay for *mtLSUrRNA* was standardized by the method described by Wakefield *et al*.^46^ and Matos *et al*.^47^. PCR assays for *DHFR* and *DHPS* genes were standardized using the published protocol of Ma and Colleagues^48^ and Costa *et al*.^49^, respectively.

PCR products were purified using a Qiagen Gel Extraction Kit (USA). Purified PCR products were directly sequenced bidirectionally using BigDye Terminator chemistry with an automated sequencer (ABI prism 310). Sequencing of PCR products was repeated twice to confirm the results. Chromatograms of PCR products were analyzed using BioEdit software version 7.1.3 with the Clustal-W alignment program. The reference sequences that were used for the analyses were M58605 for *mtLSUrRNA*, AF090368 for *DHFR*, and AY628435 for *DHPS*.

Nucleotide sequences obtained in the present study were submitted to NCBI GenBank, including wild type sequences and mutant or novel sequences. The accession numbers of novel sequences observed for the first time in the present study are as follows: *DHFR* (MG010746-MG010749; MG010751; MG010753; MG010755-MG010759; MG010761; MG010767-MG010770); *DHPS* (MG010774; MG010777; MG010779-MG010782; MG010784-MG010787; MG010789; MG010791-MG010796; MG010798; MG010799).

### Statistical analyses

Mutations and their associations with patient clinical data were studied. *p-*values were calculated by using Fisher’s exact test wherever applicable. *P-*values less than 0.05 were considered significant.

### Phylogenetic analyses

The phylogenetic tree was inferred using MEGA 7 software by the Neighbor-Joining method. The evolutionary distances were computed using the Kimura 2-parameter method. The analyses involved a total of 30 concatenated nucleotide sequences.

## References

[1] Vanek J, Jirovec O, Lukes J (1953). Interstitial plasma cell pneumonia in infants. Annales paediatrici International review of pediatrics..

[2] Gajdusek DC (1957). Pneumocystis carinii; etiologic agent of interstitial plasma cell pneumonia of premature and young infants. Pediatrics..

[3] Bakeera KS, Musoke P, Downing R, Tumwine JK (2004). Pneumocystis carinii in children with severe pneumonia at Mulago Hospital, Uganda. Annals of tropical paediatrics..

[4] Ling C (2016). Pneumocystis pneumonia in non-HIV children: A 10-year Retrospective Study. The clinical respiratory journal..

[5] Lazzerini M (2016). Mortality and its risk factors in Malawian children admitted to hospital with clinical pneumonia, 2001-12: a retrospective observational study. The Lancet Global health..

[6] Monroy-Vaca EX (2014). Genetic diversity of Pneumocystis jirovecii in colonized Cuban infants and toddlers. Infection, genetics and evolution..

[7] Badiee P, Rezapour A, Abbasian A, Foroutan HR, Jafarian H (2016). Prevalence of colonization and mitochondrial large subunit rRNA mutation of Pneumocystis jiroveci among Iranian children. Iranian journal of microbiology..

[8] Hauser PM, Francioli P, Bille J, Telenti A, Blanc DS (1997). Typing of Pneumocystis carinii f. sp. hominis by single-strand conformation polymorphism of four genomic regions. Journal of clinical microbiology..

[9] Matos O, Esteves F (2010). Pneumocystis jirovecii multilocus gene sequencing: findings and implications. Future microbiology..

[10] Phipps LM (2011). Nosocomial Pneumocystis jirovecii pneumonia: lessons from a cluster in kidney transplant recipients. Transplantation..

[11] Maitte C (2013). Multilocus sequence typing of Pneumocystis jirovecii from clinical samples: how many and which loci should be used?. Journal of clinical microbiology..

[12] Esteves F (2009). Genetic characterization of the UCS and Kex1 loci of Pneumocystis jirovecii. European journal of clinical microbiology & infectious diseases..

[13] Sassi M (2012). Outbreaks of Pneumocystis pneumonia in 2 renal transplant centers linked to a single strain of Pneumocystis: implications for transmission and virulence. Clinical infectious diseases..

[14] Miller RF, Wakefield AE (1999). Pneumocystis carinii genotypes and severity of pneumonia. Lancet..

[15] Beard CB (2000). Genetic variation in Pneumocystis carinii isolates from different geographic regions: implications for transmission. Emerging infectious diseases..

[16] Miller RF, Ambrose HE, Novelli V, Wakefield AE (2002). Probable mother-to-infant transmission of Pneumocystis carinii f. sp. hominis infection. Journal of clinical microbiology..

[17] de Boer MG (2007). An outbreak of Pneumocystis jiroveci pneumonia with 1 predominant genotype among renal transplant recipients: interhuman transmission or a common environmental source?. Clinical infectious diseases: an official publication of the Infectious Diseases Society of America..

[18] de Armas Y (2012). Low genetic diversity of Pneumocystis jirovecii among Cuban population based on two-locus mitochondrial typing. Medical mycology..

[19] Ponce, C. A. *et al*. High Prevalence of *Pneumocystis jirovecii* Dihydropteroate Synthase Gene Mutations in Patients with a First Episode of *Pneumocystis* Pneumonia in Santiago, Chile, and Clinical Response to Trimethoprim-Sulfamethoxazole Therapy. Antimicrob Agents Chemother, **61**(2). (2017). 10.1128/AAC.01290-16 PMC5278682 27855071

[20] Morris A, Norris KA (2012). Colonization by Pneumocystis jirovecii and its role in disease. Clinical microbiology reviews..

[21] Lanaspa M (2015). High prevalence of Pneumocystis jirovecii pneumonia among Mozambican children <5 years of age admitted to hospital with clinical severe pneumonia. Clin Microbiol Infect..

[22] Azoulay E (2009). Polymerase Chain Reaction for Diagnosing Pneumocystis Pneumonia in Non-HIV Immunocompromised Patients With Pulmonary Infiltrates. Chest..

[23] Azoulay E (2010). Diagnostic strategy for hematology and oncology patients with acute respiratory failure: randomized controlled trial. Am J Respir Crit Care Med.

[24] Roux A (2014). *Pneumocystis jirovecii pneumonia in patients with or without AIDS*, *France*. Emerg Infect Dis.

[25] Olsson, M., Elvin, K., Lofdahl, S. & Linder, E. Detection of *Pneumocystis carinii* DNA in sputum and bronchoalveolar lavage samples by polymerase chain reaction. *J Clin Microbiol*. **31**(2), 221en dash6 (1996). 10.1128/jcm.31.2.221-226.1993 PMC262739 8432806

[26] Pinlaor S (2004). PCR diagnosis of *Pneumocystis carinii* on sputum and bronchoalveolar immunocompromised patients. Parasitol Res..

[27] Wakefield AE (1990). Detection of Pneumocystis carinii with DNA amplification. Lancet..

[28] Gupta R (2008). Use of Different Primer Directed Sequence Amplification by Polymerase Chain Reaction for Identification of Pneumocystis jirovecii in Clinical Samples. Indian J Chest Dis Allied Sci..

[29] Martinez Lamas L (2018). Role of Pneumocystis jirovecii in patients with different pulmonary underlying condition using a nested-PCR. Rev Esp Quimioter.

[30] Sokulska M (2018). Genotyping of Pneumocystis jirovecii in colonized patients with various pulmonary diseases. Med Mycol.

[31] Das CK (2014). Use of Induced sputum to determine the prevalence of Pneumocystis jirovecii in immunocompromised children with pneumonia. Journal of tropical pediatrics..

[32] Esteves F (2010). Identification of relevant single-nucleotide polymorphisms in Pneumocystis jirovecii: relationship with clinical data. Clinical microbiology and infection: the official publication of the European Society of Clinical Microbiology and Infectious Diseases..

[33] Singh Y (2017). Circulating genotypes of Pneumocystis jirovecii and its clinical correlation in patients from a single tertiary center in India. European journal of clinical microbiology & infectious diseases: official publication of the European Society of Clinical Microbiology..

[34] Nahimana A, Rabodonirina M, Bille J, Francioli P, Hauser PM (2004). Mutations of Pneumocystis jirovecii dihydrofolate reductase associated with failure of prophylaxis. Antimicrobial agents and chemotherapy..

[35] Singh Y (2015). Molecular detection of DHFR gene polymorphisms in Pneumocystis jirovecii isolates from Indian patients. Journal of infection in developing countries..

[36] Peterson DS, Walliker D, Wellems TE (1988). Evidence that a point mutation in dihydrofolate reductase-thymidylate synthase confers resistance to pyrimethamine in falciparum malaria. Proceedings of the National Academy of Sciences of the United States of America..

[37] Dale GE (1997). A single amino acid substitution in Staphylococcus aureus dihydrofolate reductase determines trimethoprim resistance. Journal of molecular biology..

[38] Pikis A, Donkersloot JA, Rodriguez WJ, Keith JM (1998). A conservative amino acid mutation in the chromosome-encoded dihydrofolate reductase confers trimethoprim resistance in Streptococcus pneumoniae. The Journal of infectious diseases..

[39] Perna NT (2001). Genome sequence of enterohaemorrhagic *Escherichia coli* O157:H7. Nature..

[40] Lane BR (1997). Dihydropteroate synthase polymorphisms in Pneumocystis carinii. The Journal of infectious diseases..

[41] Kazanjian P (1998). Pneumocystis carinii mutations associated with sulfa and sulfone prophylaxis failures in AIDS patients. AIDS..

[42] Long Y, Zhang C, Su L, Que C (2014). Pneumocystis jirovecii dihydropteroate synthase gene mutations in a group of HIV-negative immunocompromised patients with Pneumocystis pneumonia. Experimental and therapeutic medicine..

[43] Suarez I (2017). Low prevalence of DHFR and DHPS mutations in Pneumocystis jirovecii strains obtained from a German cohort. Infection..

[44] Peters SG, Prakash UB (1987). Pneumocystis carinii pneumonia. Review of 53 cases. Am J Med..

[45] Acar J (2013). Pneumocystis jirovecii and Cytomegalovirus Co-Infection in AIDS Patients. Journal of Medical Cases. North America..

[46] Wakefield AE (1990). Amplification of mitochondrial ribosomal RNA sequences from Pneumocystis carinii DNA of rat and human origin. Molecular and biochemical parasitology..

[47] Matos O (2001). Effect of oral washes on the diagnosis of Pneumocystis carinii pneumonia with a low parasite burden and on detection of organisms in subclinical infections. European journal of clinical microbiology & infectious diseases..

[48] Ma L, Borio L, Masur H, Kovacs JA (1999). Pneumocystis carinii dihydropteroate synthase but not dihydrofolate reductase gene mutations correlate with prior trimethoprim-sulfamethoxazole or dapsone use. The Journal of infectious diseases..

[49] Costa MC (2005). Detection of Pneumocystis jirovecii dihydropteroate synthase polymorphisms in patients with Pneumocystis pneumonia. Scandinavian journal of infectious diseases..

